# Brazilian nephrologist’s knowledge of intradialytic exercise: a national survey

**DOI:** 10.1590/2175-8239-JBN-2024-0274en

**Published:** 2025-10-06

**Authors:** Monaline do Nascimento Alves Cordeiro, Juliana Rodrigues da Silva, Júlio Henrique Policarpo, Damião Ernane de Souza, Juliana Fernandes de Souza Barbosa, Patrícia Érika de Melo Marinho

**Affiliations:** 1 Universidade Federal de Pernambuco, Programa de Pós-Graduação em Fisioterapia, Recife, PE, Brazil.; 2 Instituto Brasileiro de Geografia e Estatística, Natal, RN, Brazil.

**Keywords:** Clinical Decision-Making, Cross-Sectional Studies, Nephrologists, Nephrology, Renal Insufficiency, Chronic

## Abstract

**Introduction:**

Nephrologists’ lack of knowledge regarding the benefits of intradialytic exercise (IE) may result in patients not being adequately instructed to participate in exercise programs and to become more active.

**Purpose:**

To evaluate the knowledge of Brazilian nephrologists regarding guidance and importance of IE for patients with chronic kidney disease.

**Methods:**

This was a cross-sectional survey of 16 items administered to Brazilian nephrologists actively registered with the Brazilian Nephrology Society.

**Results:**

A total of 262 nephrologists participated in the study. Most of the participants were from the southeast region (n = 124, 47.3%). Additionally, 140 nephrologists (53.4%) had more than 11 years of experience. Notably, 172 nephrologists (65.5%) reported that they had not received information about the importance of IE during their formal training. Nephrologists endorsing exercise during the intradialytic period (OR = 2.00, p = 0.03) perceive its impact on their patients (OR = 6.21, p = 0.01). With an increase in years of nephrology training, there was a decreased likelihood of being informed about IE (OR = 0.13, p < 0.01). The willingness to provide exercise guidance for CKD patients is associated with nephrologists' perception of the impact of exercise (OR 7.07, p = 0.01).

**Conclusion:**

Most nephrologists were not knowledgeable about IE, those who were knowledgeable were more likely to recommend it, and those with more years of experience tended to be less knowledgeable about IE. Having this knowledge seems to have the potential to reduce barriers that affect the practice of IE.

## Introduction

Patients on hemodialysis (HD) are often sedentary and rarely encouraged to adopt an active lifestyle, including physical activity^1,2,3,4^. In contrast, the few patients who perform intradialytic exercises (IE) have shown beneficial results in aerobic capacity, fatigue, sleep, physical fitness, and consequently quality of life^2,5,6,7,8^.

Several factors contribute to IE not being carried out. Among the barriers, the following stand out: specific conditions of the disease and patient, lack of resources and funding, and attitudes and beliefs of team members^9,10,11^. Considering that there are few exercise professionals in dialysis rooms, the responsibility for promoting and guiding exercise practice lies with the physicians and other professionals who form the minimal dialysis team^12^.

Although clinical guidelines and research recommend regular exercise for patients with chronic kidney disease (CKD), nephrologists often lack the necessary knowledge and confidence to provide appropriate guidance, resulting in the lack of counseling^13,14,15,16,17^. Factors such as lack of training, time, and knowledge to correctly prescribe exercises were also observed among nephrologists and other health professionals^15,18,19^. The lack of guidance is another barrier to physical exercise during HD^10,20,21^.

Thus, considering that most care for patients with CKD is provided by nephrologists and that medical guidelines have a great influence on treatment adherence, pharmacological or otherwise, nephrologists’ knowledge about the benefits of exercise during the intradialytic period can improve guidance on providing care for these patients, encourage participation in exercise programs, and promote a more active lifestyle^9,10,14,16^. Therefore, evaluating the information provided by Brazilian nephrologists regarding the guidance and importance of performing intradialytic exercise (IE) in patients with CKD is of considerable relevance.

## Methods

This was a cross-sectional survey with Brazilian nephrologists registered with the Brazilian Nephrology Society (BNS) conducted from February to July 2023. The study was approved by the institutional ethics committee (no. 5,882,724) in accordance with Resolution 466/12, and all individuals who participated in the study gave their informed consent. In addition, the Consensus-Based Checklist for Reporting Research Studies (CROSS) was followed. Nephrologists who lived outside Brazil or did not have an up-to-date email address were excluded^22^.

### Study Protocol

A questionnaire was prepared for this study, and consisted of two parts. The first part were questions regarding the time (years) since nephrology training, practice region (North, Northeast, Central-West, South and Southeast), and nephrology experience time (years). The second part consisted of questions relating to knowledge about the importance of physical exercise (yes/no); knowledge about the benefits of IE (yes/no/no opinion); advice and indication of IE (yes/no/no opinion); guidance on the importance of regular physical exercise for patients with CKD (yes/no); and professional which the participant believed that should guide and develop the exercise program (physiotherapist/exercise physiologist/other).

The independent variables region of operation (North, Northeast, South, Southeast and Central-west), service sector at work (public, private or both), years of training (1 to < 2 years; 2 to < 5 years; 5 to 10 years, and 11 or more years), HD experience (1 to < 2 years; 2 to < 5 years; 5 to 10 years and 11 years or more), knowledge about the physical activity level of patients with CKD (‘yes’ and ‘no’), guidance to perform physical exercise (‘yes’ and ‘no’), knowledge about exercise physical (‘yes’ and ‘no’), indication of exercise for HD patients (‘yes’, ‘no’ and ‘no opinion’), patient’s perception of physical improvement (‘yes’, ‘no’ and ‘no opinion’), professional responsible for conducting the exercise (‘physiotherapist’, ‘exercise physiologist’, ‘other’) and existence of an exercise program in HD (‘yes’, ‘no’ and ‘does not know how to inform’), as well as the dependent variables were described using absolute and percentage frequencies. The ‘exercise guidance’ (‘yes’, ‘no’ and ‘I don’t know’) and ‘knowledge about the benefits of exercise in the intradialytic period’ (‘yes’, ‘no’ and ‘I don’t know’) variables were considered dependent variables.

### Pre-testing of The Questionnaire

The prepared questionnaire was first reviewed for logic, flow, ambiguity and number of questions^23^. Then, the questionnaire was sent to 10 physiotherapists in order to investigate the properties of accuracy, clarity and objectivity, redundancy, irrelevance or poor formulation of the questions. These professionals were also asked to record the time taken to complete the questionnaire. The information obtained through the pre-test was used to improve the questionnaire.

### Sending the Form to Nephrologists

Data were collected through a questionnaire developed on the Google Forms platform and sent to the e-mails of nephrologists registered with the BSN. The e-mail contained access to a link to the form with explanations about the study, the informed consent form, and direct access to the questionnaire, if consent was provided. The e-mails were sent biweekly for a total of nine rounds of form submission. The response rate was calculated for each sending wave.

### Statistical Analysis

The data were initially computed in an Excel spreadsheet and then exported and processed in the SPSS version 22 program. Pearson’s chi-squared test was performed to check associations between variables. Variables that presented associations ≤ 0.20 were included in the multivariate model. Next, two binary logistic regression models (Models 1 and 2) were constructed to identify the factors associated with IE indication by nephrologists. We used the Enter method, retaining all preselected variables in the final model to avoid premature exclusion of potential predictors. This conservative approach aligns with methods used in similar clinical studies^24^.

All analyzes were performed considering a p-value < 0.05 (two-tailed).

## Results

**Figure 1:**
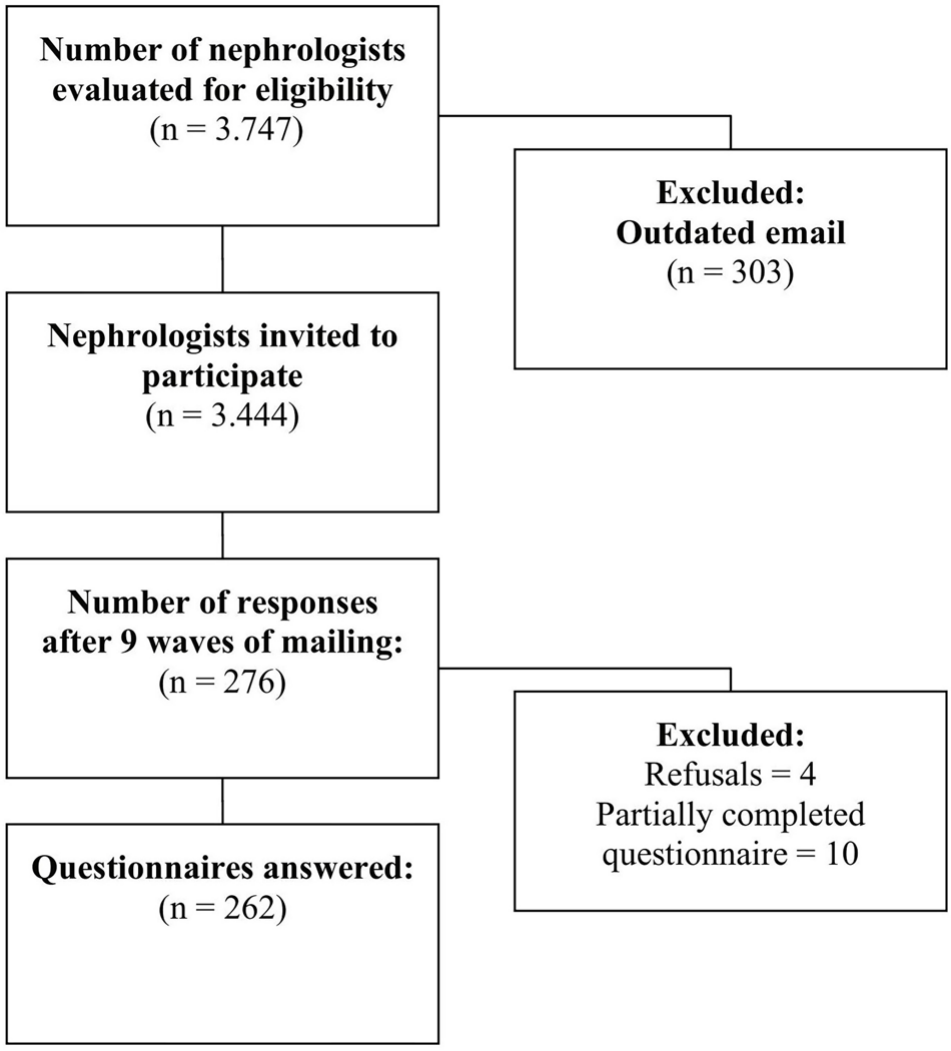
Flowchart of the recruitment of questionaries responses.

The study population consisted of 3,747 Brazilian nephrologists registered with the BSN. Of these, 303 had outdated e-mails, representing a total of 3,444 eligible nephrologists. A total of 276 nephrologists opened the invitation email (8.3%), however four did not accept to participate and 10 questionnaires were partially answered, resulting in 262 forms actually answered (7.6%). The final sample for this study consisted of 256 nephrologists (Figure 1). The participation rate in this study (7.6%) was within the range commonly observed for studies conducted via email, which ranges from 7 to 13%.

The majority of professionals were concentrated in Southeast region (n = 124, 47.3%), followed by the Northeast (n = 53, 20.2%), South (n = 38, 14.5%), Central-West (n = 28, 10.7%), and North (n = 19, 7.3%) regions. Most nephrologists had 11 years or more of training (n = 140, 53.4%), 63 (24.1%) between 1 and 5 years, and 59 of them (22.5%) between 5 and 10 years. Concerning work sector, 57.3% performed activities in the private and public sectors (n = 150), 57 in the private only (21.8%), and 55 in the public only (21.0%).

Regarding IE knowledge, 155 (59.2%) of nephrologists reported that their patients perform some type of physical exercise, 224 (85.5%) usually asked their patients about the level of physical activity, 243 (92.7%) reported providing guidance for physical activity, 239 (91.2%) observed clinical and functional benefits in patients who exercised, and 108 (41.2%) considered the physiotherapist a qualified professional to develop an exercise program for patients with CKD.

Although 172 (65.5%) nephrologists who participated in the study responded that they did not receive information about the importance IE during their formal training, 242 (92.4%) recognized that exercise is beneficial. This recognition translates into the indication for it to be performed during HD (n = 157, 59.9%). Although the majority (n = 243, 92.7%) recognized that IE should be performed, 70.6% (n = 185) reported that there was no exercise program in the sectors where they worked. For those who responded that there was an exercise professional available at the service, 22.9% (n = 60) reported the physiotherapist as being responsible for conducting the program (Table 1).

**Table 1:** Nephrologists’ knowledge about the practice of therapeutic exercises in the intradialytic period

n = 262%		
Information about the importance of IE during training
No	172	65.60
Yes	90	34.40
IE program is beneficial for patients		
No	20	7.60
Yes	242	92.40
Indication of IE for patients		
No	105	40.10
Yes	157	59.90
IE program improves patients’ physical activity level		
No	41	15.60
Yes	221	84.40
IE program supervised by a qualified professional		
No	19	7.30
Yes	243	92.70
There is an IE program in the service where you work		
No	192	73.30
Yes	70	26.70
Professional responsible for developing the IE program		
Physiotherapist	60	22.90
Exercise physiologist	7	2.70
Other	3	1.20

The factor associated with exercise guidance for patients with CKD was the nephrologist perception of the benefit of exercise [OR 7.07 (95% CI 2.17 to 23.04), p < 0.01].

Nephrologists recommending exercise in the intradialytic period (OR = 2.00, p = 0.03) are those who believe in the beneficial impact of exercise on their patients (OR = 6.21, p = 0.01). In addition, the longer the time since nephrology training, the less likely the participant was to have received information about IE (OR = 0.13, p < 0.01) (Table 2).

**Table 2:** Factors associated with formal training on the importance of IE as a nephrologist

	OR	95%CI	p-value
Recommend intradialytic exercise			
No	Reference	Reference	Reference
Yes	2.00	(1.06 to 3.76)	0.03
Exercise improves the condition			
No	Reference	Reference	Reference
Yes	6.21	(1.34 to 28.76)	0.01
Time since graduation			
< 2 years	Reference	Reference	Reference
2 to 5 years	0.36	(0.11 to 1.19)	0.09
5 to 10 years	0.33	(0.10 to 1.08)	0.05
11 years or more	0.13	(0.04 to 0.38)	< 0.01

Note – Nagelkerke R^2^ = 0.17

## Discussion

This study found that physical activity was most frequently recommended by nephrologists who recognize its importance. However, most of them did not receive formal education about IE during their training and few reported the presence of an exercise professional in the services where they work. Furthermore, those who were informed about the importance of IE were more likely to recommend exercise, while professionals with more time since training were less likely to have received formal training on this subject.

The recommendation of IE for patients with CKD was associated with nephrologist perception that exercise contributes to the improvement of the clinical condition of patients. This view aligns with the findings of Taryana et al.^14^ and Bennett et al.^25^, which revealed that nephrologists agree that regular exercise offers health benefits across all stages of CKD. Furthermore, studies show that exercising during hemodialysis improve functional, physiological, and psychological adaptations, is safe and optimizes patients’ time by allowing them to exercise concurrently with HD^23,26^.

Previous studies that assessed the motivations and barriers to IE revealed that factors such as low motivation, discomfort to nurses, and safety concerns are considered by patients as barriers to performing exercise^9,10,18,19,27^. Informing professionals who work in the HD service about IE for can minimize some of these barriers^15^.

Our study found that more than half of the nephrologists did not receive information about IE in their training. This situation is similar for other professionals in the essential multidisciplinary teams required in HD rooms, indicating a general lack of knowledge on this topic. While we recognize that simply acquiring this knowledge is not sufficient to address the sedentary behavior observed in dialysis centers, we consider it essential for enhancing the guidance provided to patients regarding exercise^14^.

According to the observed results, the probability of receiving information about exercise during nephrology training was lower for nephrologists with greater experience in the area. Although other factors were not investigated in this study, we assume that despite the favorable evidence of IE, it is not a topic which is discussed and encouraged during nephrologist training^12^. Therefore, updates and workshops could positively impact the low rates of IE counseling, particularly among older nephrologists.

The lack of information on exercise and its benefits during nephrology training may lead to gaps in the care of HD patients, who require complex treatment involving not only dialysis and drug therapy but also multidisciplinary interventions, such as nutritional and psychological monitoring, lifestyle counseling, and guidance on physical activity^10,12,28^.

Although most HD services did not have physiotherapists, participants highlighted the importance of having a qualified professional to supervise IE programs. In services where exercise programs were available, physiotherapists were usually responsible for their development^29^. The lack of regulation and no legal requirement to include physiotherapists on HD teams may explain the absence or low number of these professionals in dialysis centers.

Parker et al.^30^ demonstrated that involvement of physical therapists in HD contributed to an increase in regular exercise practice, and observed that patient adherence more than doubled when a trained professional was present to perform the exercise. Johansen et al.^31^ suggested that the presence of these professionals could help reduce the barriers identified by physicians such as lack of time and confidence to provide guidance on the subject, since other professionals would also be involved in reducing patients inactivity.

Our data also demonstrated that nephrologists with less time since nephrology training were more likely to advise patients to exercise compared to those with more years of experience, similar to the study by Silva and Marinho^32^, where nephrologists with less than two years since graduating were ten times more likely to prescribe exercises. It is possible that nephrologists with less time since graduating had the opportunity to establish contact with exercise programs in HD centers, a growing but recent practice in nephrology services^13^.

We recognize that our study had some limitations, particularly the difficulty in obtaining updated email addresses for all nephrologists and the low response rate, despite sending out nine rounds of emails. However, a key strength lies in the participation of nephrologists from all regions of Brazil providing a comprehensive national overview of their nephrology training and their knowledge regarding IE.

This study has important clinical implications. Understanding nephrologists’ knowledge regarding IE and its recommendation to patients with CKD is a crucial step in developing strategies to enhance counseling and adherence to exercise in hemodialysis. Additionally, even if IE is not routinely recommended by nephrologists, it is essential that they be informed about this topic. This knowledge will allow them to address other barriers, such as a lack of funding and qualified personnel.

In conclusion, the current study revealed that most nephrologists did not receive information about IE during their training, and those who did were more likely to recommend it, as exercise improves the clinical condition of patients. On the other hand, professionals with more experience in the field were less likely to have received IE information. Therefore, acquiring this knowledge is important as it may help reduce the barriers that exist in the practice of IE.

## Data Availability

Data is available upon reasonable request to the corresponding author.

## Supplementary Material

The following online material is available for this article:

Knowledge of Brazilian nephrologists about intradialytic exercise: A national survey.
