# Supplementary material for: Brazilian nephrologist’s knowledge of intradialytic exercise: a national survey
Source: J Bras Nefrol. 2025 Oct 6;48(1):e20240274. doi: 10.1590/2175-8239-JBN-2024-0274en (PMC12534031; doi:10.1590/2175-8239-JBN-2024-0274en)
Supplement: Supplementary file 1 [file 2175-8239-jbn-48-1-e20240274-suppl1.pdf]

Braz. J. Nephrol.
https://doi.org/10.1590/2175-8239-JBN-2024-0274en

Supplementary Material to "Brazilian nephrologist's knowledge of intradialytic exercise: A national survey"

Knowledge of Brazilian nephrologists about intradialytic exercise: A national survey

We invite you to participate as a volunteer in the research study entitled "Knowledge About Intradialytic Exercise: A Survey of Brazilian Nephrologists." Your participation is crucial for assessing your understanding of the importance of recommending exercise programs during the intradialytic phase, which should be developed by a qualified health professional. We thank you in advance for your time and contribution.

Sincerely,

The Research Team

ATTENTION:

This questionnaire consists of 16 questions.

Before answering, it is important to understand the following definitions:

- **ACTIVE:** An individual who engages in physical activity five or more days a week for a total of 150 minutes or more each week.
- **SEDENTARY:** An individual who does not participate in any physical activity for at least ten continuous minutes during the week.
- **PHYSICAL ACTIVITY:** Any movement of the body produced by skeletal muscles that results in an increase in energy expenditure above resting levels.
- **PHYSICAL EXERCISE:** A structured form of physical activity that involves a planned sequence of systematically repeated movements, with a defined frequency, duration, and intensity, aimed at improving or maintaining one or more components of health-related physical fitness.

1- How long ago did you finish your Nephrology training?

( ) 1 to 2 years

( ) 2 to 5 years

( ) 5 to 10 years

( ) 11 or more years

2- How long have you been working as a nephrologist monitoring hemodialysis patients?
3- ( ) 1 to 2 years
4- ( ) 2 to 5 years
5- ( ) 5 to 10 years
6- ( ) 11 or more years

3- In which region do you currently work as a Nephrologist?

( ) North

( ) Northeast

( ) South

( ) Southeast

( ) Midwest

3- Do the patients you follow with conservative stage CKD do any physical exercise?

( ) Yes

( ) No

( ) I don't know

4- In which service do you currently provide care as a nephrologist?

( ) Private

( ) Public

( ) Both

5- Is there a physiotherapist in the hemodialysis team in the private sector?

( ) Yes

( ) No

( ) I don't know

6- Is there a physiotherapist in the hemodialysis team in public service?

( ) Yes

( ) No

( ) I don't know

7- Do you know if there is a physiotherapist in the hemodialysis team where you work?

( ) Yes

( ) No

( ) I don't know

8- Do you typically inquire about your patients' physical activity level, whether active or sedentary?

( ) Yes

( ) No

9- Do you advise patients to exercise?

( ) Yes

( ) No

10- Could you please specify which service you refer patients to?

( ) Gym

( ) Physiotherapy clinic

( ) Others

11- Which healthcare professional is best qualified to develop and implement an exercise program for a patient during the conservative CKD stage?

( ) Physiotherapist

( ) Exercise physiologist

( ) Other

12- Do you notice any clinical and functional differences between patients who exercise and those who do not?

( ) Yes

( ) No

( ) I don’t know

13- Did you receive information about the importance of intradialytic exercise during your nephrology training?

( ) Yes

( ) No

14- Do you think a physical exercise program is beneficial for patients during dialysis?

( ) Yes

( ) No

( ) I don’t know

15- Do you recommend intradialytic exercise for patients who are stable during hemodialysis?

( ) Yes

( ) No

( ) I don’t know

16- Do you believe that an intradialytic exercise program improves the physical activity level of patients?

( ) Yes

( ) No

( ) I don’t know

17- Should there be a supervised intradialytic exercise program for these patients?

( ) Yes

( ) No

( ) I don’t know

18- Is there an ongoing intradialytic exercise program available in your service, whether public, private, or both?

( ) Yes

( ) No

( ) I don’t know

19- Who is responsible for developing the intradialytic exercise program at your workplace?

( ) Physiotherapist

( ) Exercise physiologist

( ) Physician

( ) Other
