# Supplementary material for: Brazilian nephrologist’s knowledge of intradialytic exercise: a national survey
Source: J Bras Nefrol. 2025 Oct 6;48(1):e20240274. doi: 10.1590/2175-8239-JBN-2024-0274en (PMC12534031; doi:10.1590/2175-8239-JBN-2024-0274en)
Supplement: Translation

# Translation: Conhecimento de nefrologistas brasileiros sobre exercícios intradiáliticos: uma pesquisa nacional

**DOI:** 10.1590/2175-8239-JBN-2024-0274pt

**Authors:** Monaline do Nascimento Alves Cordeiro, Juliana Rodrigues da Silva, Júlio Henrique Policarpo, Damião Ernane de Souza, Juliana Fernandes de Souza Barbosa, Patrícia Érika de Melo Marinho

**Affiliations:** 1 Universidade Federal de Pernambuco, Programa de Pós-Graduação em Fisioterapia, Recife, PE, Brasil.; 2 Instituto Brasileiro de Geografia e Estatística, Natal, RN, Brasil.

**Keywords:** Tomada de Decisão Clínica, Estudos Transversais, Nefrologistas, Nefrologia, Insuficiência Renal Crônica

## Abstract

**Introdução:**

A falta de conhecimento dos nefrologistas sobre benefícios do exercício intradialítico (EI) pode resultar na ausência de orientações adequadas aos pacientes para que participem de programas de exercícios, tornando-se mais ativos.

**Objetivo:**

Avaliar o conhecimento de nefrologistas brasileiros quanto às orientações e à importância do EI para pacientes com doença renal crônica.

**Métodos:**

Estudo transversal com a aplicação de questionário de 16 itens a nefrologistas brasileiros com registro ativo na Sociedade Brasileira de Nefrologia.

**Resultados:**

Um total de 262 nefrologistas participaram do estudo. A maioria era da região Sudeste (n = 124; 47,3%). Além disso, 140 nefrologistas (53,4%) tinham mais de 11 anos de experiência. Notavelmente, 172 nefrologistas (65,5%) relataram não ter recebido informações sobre a importância do EI durante sua formação acadêmica. Nefrologistas que recomendam a prática de exercícios durante o período intra­diálitico (OR = 2,00; p = 0,03) percebem seu impacto nos pacientes (OR = 6,21; p = 0,01). Com o aumento dos anos de formação em nefrologia, observou-se menor probabilidade de receber informações sobre EI (OR = 0,13; p < 0,01). A disposição em fornecer orientação sobre exercícios para pacientes com DRC associa-se à percepção dos nefrologistas quanto ao impacto dos exercícios (OR 7,07; p = 0,01).

**Conclusão:**

A maioria dos nefrologistas não possuía conhecimento sobre EI, aqueles que possuíam eram mais propensos a recomendá-lo, e aqueles com mais anos de experiência tendiam a ter menos conhecimento sobre EI. Possuir esse conhecimento parece ter o potencial de reduzir as barreiras que afetam a prática do EI.

## Introdução

Pacientes em hemodiálise (HD) frequentemente apresentam comportamento sedentário e raramente são incentivados a adotar um estilo de vida ativo, incluindo a prática de atividades físicas^1–4^. Em contraste, os poucos pacientes que realizam exercícios intradialíticos (EI) apresentaram resultados benéficos na capacidade aeróbica, fadiga, sono, aptidão física e, consequentemente, qualidade de vida^2,5–8^.

Diversos fatores contribuem para que o EI não seja realizado. Entre as barreiras, destacam-se: condições específicas da doença e do paciente, escassez de recursos e financiamento, além de atitudes e crenças de membros das equipes^9–11^. Considerando que há poucos profissionais de educação física nos centros de diálise, a responsabilidade pela promoção e orientação da prática de exercícios recai sobre os médicos e demais profissionais que compõem a equipe mínima de diálise^12^.

Embora as diretrizes clínicas e pesquisas recomendem a prática regular de atividade física para pacientes com doença renal crônica (DRC), os nefrologistas muitas vezes não possuem o conhecimento e a confiança necessários para fornecer orientações adequadas, resultando na ausência de aconselhamento^13–17^. Fatores como falta de treinamento, tempo e conhecimento para prescrever exercícios corretamente também foram observados entre nefrologistas e outros profissionais de saúde^15,18,19^. A falta de orientação é outra barreira à prática de atividade física durante a HD^10,20,21^.

Dessa forma, considerando que a maior parte dos cuidados aos pacientes com DRC é prestada por nefrologistas e que as diretrizes médicas exercem grande influência na adesão ao tratamento, farmacológico ou não, o conhecimento dos nefrologistas sobre os benefícios dos exercícios durante o período intradiálito pode melhorar a orientação no cuidado desses pacientes, incentivar a participação em programas de exercícios e promover um estilo de vida mais ativo^9,10,14,16^. Portanto, avaliar as informações fornecidas por nefrologistas brasileiros a respeito da orientação e da importância da prática de exercícios intradialíticos (EI) em pacientes com DRC é de considerável relevância.

## Métodos

Este foi um estudo transversal com nefrologistas brasileiros registrados na Sociedade Brasileira de Nefrologia (SBN) realizado entre fevereiro e julho de 2023. O estudo foi aprovado pelo comitê de ética institucional (nº 5.882.724) em conformidade com a Resolução 466/12, e todos os indivíduos que participaram do estudo deram seu consentimento informado. Além disso, foi seguido o *Consensus-Based Checklist for Reporting Research Studies* (CROSS). Foram excluídos os nefrologistas que residiam fora do Brasil ou que não possuíam um endereço de e-mail atualizado^22^.

### Protocolo do Estudo

Foi elaborado um questionário para este estudo, composto por duas partes. A primeira parte continha questões relativas ao tempo (em anos) desde a formação em nefrologia, região de atuação (Norte, Nordeste, Centro-Oeste, Sul e Sudeste) e tempo de experiência em nefrologia (em anos). A segunda parte consistiu em questões relacionadas ao conhecimento sobre a importância do exercício físico (sim/não); conhecimento sobre os benefícios do EI (sim/não/sem opinião); orientação e indicação de EI (sim/não/sem opinião); orientação sobre a importância da prática regular de exercícios físicos para pacientes com DRC (sim/não); e profissional que o participante considerava como adequado para orientar e desenvolver o programa de exercícios (fisioterapeuta/fisiologista do exercício/outro).

As variáveis independentes – região de atuação (Norte, Nordeste, Sul, Sudeste e Centro-Oeste), setor de atuação (público, privado ou ambos), tempo de formação (1 a < 2 anos; 2 a < 5 anos; 5 a 10 anos e 11 anos ou mais), experiência em HD (1 a < 2 anos; 2 a < 5 anos; 5 a 10 anos e 11 anos ou mais), conhecimento sobre o nível de atividade física dos pacientes com DRC (“sim” e “não”), orientação para a prática de exercícios físicos (“sim” e “não”), conhecimento sobre exercícios físicos (“sim” e “não”), indicação de exercícios para pacientes em HD (“sim”, “não” e “sem opinião”), percepção de melhora física do paciente (“sim”, “não” e “sem opinião”), profissional responsável pela condução do exercício (“fisioterapeuta”, “fisiologista do exercício”, “outro”) e existência de um programa de exercícios em HD (“sim”, “não” e “não sabe informar”) –, bem como as variáveis dependentes, foram descritas utilizando frequências absolutas e percentuais. As variáveis “orientação sobre exercícios” (“sim”, “não” e “não sei”) e “conhecimento sobre os benefícios dos exercícios no período intradiálitico” (“sim”, “não” e “não sei”) foram consideradas variáveis dependentes.

### Pré-teste do Questionário

O questionário elaborado foi inicialmente revisado quanto à lógica, fluidez, ambiguidade e número de questões^23^. Em seguida, o instrumento foi enviado a 10 fisioterapeutas a fim de investigar as propriedades de precisão, clareza e objetividade, redundância, irrelevância ou formulação inadequada das questões. Esses profissionais também foram solicitados a registrar o tempo necessário para preenchimento do questionário. As informações obtidas por meio do pré-teste foram utilizadas para aprimorar o questionário.

### Envio do Formulário aos Nefrologistas

Os dados foram coletados por meio de um questionário desenvolvido na plataforma *Google Forms* e enviado aos e-mails dos nefrologistas registrados na SBN. A mensagem continha o acesso a um link para o formulário com explicações sobre o estudo, o termo de consentimento informado e acesso direto ao questionário, caso o consentimento fosse fornecido. Os e-mails foram encaminhados quinzenalmente, totalizando nove rodadas de envio. A taxa de resposta foi calculada para cada uma das rodadas.

### Análise Estatística

Os dados foram inicialmente calculados em uma planilha do Excel e, em seguida, exportados e processados no programa SPSS versão 22. O teste qui-quadrado de Pearson foi realizado para verificar associações entre as variáveis. As variáveis que apresentaram associações ≤ 0,20 foram incluídas no modelo multivariado. Em seguida, foram construídos dois modelos de regressão logística binária (Modelos 1 e 2) para identificar os fatores associados à indicação de EI pelos nefrologistas. Foi utilizado o método *Enter*, mantendo todas as variáveis pré-selecionadas no modelo final para evitar a exclusão prematura de potenciais preditores. Essa abordagem conservadora está em conformidade com os métodos empregados em estudos clínicos semelhantes^24^.

Todas as análises foram realizadas considerando um valor de p < 0,05 (bicaudal).

## Resultados

A população do estudo foi composta por 3.747 nefrologistas brasileiros registrados na SBN. Destes, 303 apresentavam e-mails desatualizados, resultando em um total de 3.444 nefrologistas elegíveis. No total, 276 nefrologistas abriram o e-mail convite (8,3%); contudo, quatro não aceitaram participar e 10 questionários foram respondidos parcialmente, resultando em 262 formulários efetivamente respondidos (7,6%). A amostra final para este estudo consistiu em 256 nefrologistas (Figura 1). A taxa de participação (7,6%) ficou dentro da faixa comumente observada para estudos realizados por e-mail, que varia entre 7% e 13%.

**Figura 1:** Flowchart of the recruitment of questionaries responses.

A maioria dos profissionais estava concentrada na região Sudeste (n = 124; 47,3%), seguida pelo Nordeste (n = 53; 20,2%), Sul (n = 38; 14,5%), Centro-Oeste (n = 28; 10,7%) e Norte (n = 19; 7,3%). A maior parte dos nefrologistas tinha 11 anos ou mais de formação (n = 140; 53,4%), 63 (24,1%) entre 1 e 5 anos e 59 (22,5%) entre 5 e 10 anos. Quanto ao setor de atuação, 57,3% exerciam atividades nos setores privado e público (n = 150), 57 somente no setor privado (21,8%) e 55 exclusivamente no setor público (21,0%).

Em relação ao conhecimento sobre EI, 155 (59,2%) nefrologistas relataram que seus pacientes realizam algum tipo de atividade física, 224 (85,5%) costumam questionar seus pacientes sobre o nível de atividade física, 243 (92,7%) relataram fornecer orientações para a prática de exercícios, 239 (91,2%) observaram benefícios clínicos e funcionais em pacientes que praticavam exercícios e 108 (41,2%) consideraram o fisioterapeuta um profissional qualificado para desenvolver um programa de exercícios para pacientes com DRC.

Embora 172 (65,5%) nefrologistas que participaram do estudo tenham relatado não ter recebido informações sobre a importância do EI durante sua formação acadêmica, 242 (92,4%) reconheceram que o exercício é benéfico. Esse reconhecimento se traduz na indicação de sua realização durante a HD (n = 157; 59,9%). Ainda que a maioria (n = 243; 92,7%) tenha reconhecido que o EI deve ser realizado, 70,6% (n = 185) relataram a inexistência de um programa de exercícios nos setores em que atuavam. Entre aqueles que responderam haver um profissional de exercícios disponível no serviço, 22,9% (n = 60) apontaram o fisioterapeuta como responsável pela condução do programa (Tabela 1).

**Tabela 1:** Conhecimento dos nefrologistas sobre a prática de exercícios terapêuticos no período intradiálitico

n = 262%		
Informações sobre a importância do EI durante a formação
Não	172	65,60
Sim	90	34,40
O programa de EI é benéfico para os pacientes		
Não	20	7,60
Sim	242	92,40
Indicação de EI para pacientes		
Não	105	40,10
Sim	157	59,90
O programa de EI melhora o nível de atividade física dos pacientes		
Não	41	15,60
Sim	221	84,40
Programa de EI supervisionado por um profissional qualificado		
Não	19	7,30
Sim	243	92,70
Existe um programa de EI no serviço onde você atua		
Não	192	73,30
Sim	70	26,70
Profissional responsável pelo desenvolvimento do programa de EI		
Fisioterapeuta	60	22,90
Fisiologista do exercício	7	2,70
Outro	3	1,20

O fator associado à orientação sobre exercícios para pacientes com DRC foi a percepção do nefrologista quanto aos benefícios da atividade física [OR 7,07 (IC 95% 2,17 a 23,04; p < 0,01]. Nefrologistas que recomendam exercícios no período intradiálitico (OR = 2,00; p = 0,03) são aqueles que acreditam no impacto benéfico dos exercícios em seus pacientes (OR = 6,21; p = 0,01). Além disso, quanto mais tempo desde a formação em nefrologia, menor a probabilidade de o participante ter recebido informações sobre EI (OR = 0,13; p < 0,01) (Tabela 2).

**Tabela 2:** Fatores associados ao treinamento formal sobre a importância do EI na formação do nefrologista

	OR	IC95%	Valor de p
Recomendar exercícios intradialíticos			
Não	Referência	Referência	Referência
Sim	2,00	(1,06 a 3,76)	0,03
O exercício físico melhora a condição			
Não	Referência	Referência	Referência
Sim	6,21	(1,34 a 28,76)	0,01
Tempo desde a graduação			
< 2 anos	Referência	Referência	Referência
2 a 5 anos	0,36	(0,11 a 1,19)	0,09
5 a 10 anos	0,33	(0,10 a 1,08)	0,05
11 anos ou mais	0,13	(0,04 a 0,38)	< 0,01

Nota – Nagelkerke R^2^ = 0,17

## Discussão

Este estudo constatou que a atividade física foi mais frequentemente recomendada por nefrologistas que reconhecem sua importância. No entanto, a maioria deles não recebeu educação formal sobre EI durante sua formação e poucos relataram a presença de um profissional de exercícios físicos nos serviços onde atuam. Além disso, aqueles que receberam informações sobre a importância do EI apresentaram maior probabilidade de recomendar a prática de exercícios, enquanto os profissionais com mais tempo de formação apresentaram menor probabilidade de ter recebido treinamento formal sobre o tema.

A recomendação de EI para pacientes com DRC esteve associada à percepção dos nefrologistas de que o exercício contribui para a melhora do quadro clínico dos pacientes. Essa opinião está em consonância com os achados de Taryana et al.^14^ e Bennett et al.^25^, que demonstraram que nefrologistas concordam que a prática regular de exercícios traz benefícios à saúde em todos os estágios da DRC. Além disso, estudos revelam que a prática de exercícios durante a hemodiálise melhora as adaptações funcionais, fisiológicas e psicológicas, é segura e otimiza o tempo dos pacientes ao permitir que eles se exercitem simultaneamente à HD^23,26^.

Estudos anteriores que avaliaram as motivações e barreiras ao EI revelaram que fatores como baixa motivação, desconforto por parte da equipe de enfermagem e preocupações com a segurança são considerados pelos pacientes como obstáculos à prática de exercícios^9,10,18,19,27^. Informar os profissionais que atuam no serviço de HD sobre o EI pode minimizar algumas dessas barreiras^15^.

Nosso estudo constatou que mais da metade dos nefrologistas não recebeu informações sobre EI durante sua formação. Essa situação é semelhante para outros profissionais das equipes multidisciplinares essenciais nas salas de HD, indicando uma carência generalizada de conhecimento sobre o tema. Embora reconheçamos que a simples aquisição desse conhecimento não seja suficiente para lidar com o comportamento sedentário observado nos centros de diálise, consideramos que ele é essencial para aprimorar a orientação oferecida aos pacientes em relação à prática de exercícios^14^.

De acordo com os resultados observados, a probabilidade de receber informações sobre exercícios durante a formação em nefrologia foi menor para nefrologistas com maior tempo de experiência na área. Embora outros fatores não tenham sido investigados neste estudo, presume-se que, apesar das evidências favoráveis do EI, este não seja um tema discutido e incentivado durante a formação dos nefrologistas^12^. Portanto, atualizações e workshops poderiam impactar positivamente as baixas taxas de orientação sobre EI, especialmente entre nefrologistas mais velhos.

A falta de informações sobre exercícios físicos e seus benefícios durante a formação em nefrologia pode levar a lacunas no cuidado de pacientes em HD, os quais demandam tratamento complexo envolvendo não apenas diálise e terapia medicamentosa, mas também intervenções multidisciplinares, como acompanhamento nutricional e psicológico, aconselhamento sobre estilo de vida e orientação sobre atividade física^10,12,28^.

Embora a maioria dos serviços de HD não contasse com fisioterapeutas, os participantes destacaram a importância de ter um profissional qualificado para supervisionar os programas de EI. Nos serviços em que tais programas estavam disponíveis, os fisioterapeutas eram normalmente responsáveis pelo seu desenvolvimento^29^. A ausência de regulamentação e de requisitos legais para a inclusão de fisioterapeutas nas equipes de HD pode explicar a falta ou o baixo número desses profissionais nos centros de diálise.

Parker et al.^30^ demonstraram que o envolvimento de fisioterapeutas na HD contribuiu para um aumento na prática regular de exercícios e observaram que a adesão dos pacientes mais que dobrou com a presença de um profissional treinado para conduzir os exercícios. Johansen et al.^31^ sugeriram que a presença desses profissionais poderia ajudar a reduzir as barreiras identificadas pelos médicos, como falta de tempo e confiança para fornecer orientações sobre o tema, uma vez que outros profissionais também estariam envolvidos na redução da inatividade dos pacientes.

Nossos dados também demonstraram que os nefrologistas com menos tempo de formação em nefrologia apresentaram maior probabilidade de orientar seus pacientes a praticar exercícios físicos, em comparação àqueles com mais anos de experiência, semelhante ao observado no estudo de Silva e Marinho^32^, no qual nefrologistas com menos de dois anos de formação apresentaram dez vezes mais probabilidade de prescrever exercícios. É possível que os nefrologistas com menos tempo de formação tenham tido a oportunidade de estabelecer contato com programas de exercícios físicos em centros de HD, uma prática em crescimento, porém recente, nos serviços de nefrologia^13^.

Reconhecemos que nosso estudo apresentou algumas limitações, particularmente a dificuldade em obter endereços de e-mail atualizados de todos os nefrologistas e a baixa taxa de resposta, apesar do envio de nove rodadas de mensagens. No entanto, um ponto forte fundamental reside na participação de nefrologistas de todas as regiões do Brasil, proporcionando uma visão nacional abrangente de sua formação em nefrologia e seus conhecimentos sobre EI.

Este estudo possui implicações clínicas importantes. Compreender o conhecimento dos nefrologistas a respeito do EI e suas recomendações aos pacientes com DRC constitui uma etapa crucial no desenvolvimento de estratégias para aprimorar o aconselhamento e a adesão aos exercícios físicos na hemodiálise. Além disso, mesmo que o EI não seja recomendado rotineiramente pelos nefrologistas, é essencial que eles estejam informados sobre o tema. Esse conhecimento lhes permitirá abordar outras barreiras, como a falta de financiamento e de pessoal qualificado.

Em conclusão, o presente estudo revelou que a maioria dos nefrologistas não recebeu informações sobre EI durante sua formação, e aqueles que receberam apresentaram maior probabilidade de recomendá-la, uma vez que o exercício melhora o quadro clínico dos pacientes. Por outro lado, profissionais com mais tempo de experiência na área apresentaram menor probabilidade de ter recebido informações sobre EI. Portanto, a aquisição desse conhecimento é importante, pois pode contribuir para reduzir as barreiras existentes na prática do EI.

## Data Availability

Os dados estão disponíveis mediante solicitação razoável ao autor correspondente.

## Material Suplementar

O seguinte material online está disponível para o presente artigo

Knowledge of Brazilian nephrologists about intradialytic exercise: A national survey
